# Synergistic Enhancement of WO_3_@Co_3_O_4_ Layered Supercapacitors via PAA-Directed Electrodeposition: A Comparative Polymer Strategy with HMTA Surfactant

**DOI:** 10.3390/mi17040407

**Published:** 2026-03-26

**Authors:** Pritam J. Morankar, Chan-Wook Jeon

**Affiliations:** School of Chemical Engineering, Yeungnam University, 280 Daehak-ro, Gyeongsan 712-749, Republic of Korea; pritam.nanoworld@gmail.com

**Keywords:** WO_3_@Co_3_O_4_, polyacrylic acid, electrodeposition: layered electrode, pseudocapacitive behavior

## Abstract

In this study, a novel layered WO_3_@Co_3_O_4_ composite electrode was synthesized via a controlled electrodeposition method employing different surfactants to finely tune its nanostructure. The incorporation of polyacrylic acid (PAA) surfactant yielded an optimized P-W@Co electrode with a hierarchical porous morphology and reduced crystallite size, markedly enhancing electroactive site exposure and electron transport. Structural analyses confirmed the amorphous nature of WO_3_ and crystalline spinel Co_3_O_4_ phases forming an integrated composite architecture. Electrochemical characterizations in a three-electrode system revealed that the P-W@Co electrode exhibited superior pseudocapacitive behavior, with an areal capacitance of 11.70 F/cm^2^ at 20 mA/cm^2^ and excellent rate capability, retaining 80% capacitance at 40 mA/cm^2^. Kinetic studies demonstrated enhanced diffusion-controlled charge storage attributed to improved ion accessibility and charge transfer kinetics. To evaluate practical feasibility, asymmetric supercapacitor devices incorporating P-W@Co as the positive electrode coupled with activated carbon as the negative electrode were fabricated. This device showcased a widened operational voltage (1.5 V), outstanding areal capacitance (211 mF/cm^2^), and energy density (0.066 mWh/cm^2^). Importantly, the device exhibited exceptional cycling stability, retaining 81.8% capacitance after 7000 cycles. This work signifies a major advancement in surfactant-mediated design of WO_3_@Co_3_O_4_ layered electrodes for scalable, high-performance supercapacitor applications, combining structural stability, enhanced conductivity, and multifaceted charge storage mechanisms.

## 1. Introduction

The growing concerns over global warming and the environmental consequences of fossil fuel consumption have intensified the push toward clean and sustainable energy solutions [1,2,3]. As the world races to mitigate carbon emissions and transition to greener alternatives, the demand for efficient and reliable energy storage systems has surged, especially for applications in electric vehicles, portable electronics, and smart grids [4,5]. In this context, supercapacitors (SCs) have garnered significant attention as next-generation energy storage devices, effectively bridging the gap between conventional dielectric capacitors and rechargeable batteries. Their inherent advantages, including rapid charge–discharge capability, high power density, and outstanding cycling stability, make them highly attractive for modern energy storage demands [6,7,8,9]. However, despite these strengths, SCs are still hampered by their relatively low energy density, which continues to limit their widespread adoption in real-world applications [10]. The electrochemical performance of SCs is largely governed by the physicochemical properties of the electrode materials [11,12]. In this context, transition metal oxides (TMOs) have attracted considerable attention due to their pseudocapacitive charge storage behavior, intrinsic chemical stability, and high theoretical capacitance values [13]. Among the diverse range of TMOs, tungsten trioxide (WO_3_) stands out as a particularly promising candidate. The unique attributes of WO_3_, including its multiple accessible oxidation states (W^6+^/W^5+^/W^4+^), open crystal framework, and efficient ion and electron transport pathways, facilitate its application as an intercalation-type electrode material. These properties enable WO_3_ to deliver stable electrochemical performance in aqueous electrolytes, often outperforming other TMOs such as MnO_2_ and NiO in terms of cycling stability [14]. Despite these advantages, the intrinsic limitations of WO_3_, namely its low electrical conductivity and susceptibility to structural degradation during long-term cycling, have hindered its practical application in high-performance SCs [15]. In response to these challenges, a growing body of research has explored the potential of WO_3_-based materials to enhance electrochemical properties through nanoscale engineering and composite formation. For instance, Sun et al. demonstrated an asymmetric supercapacitor (ASC) employing WO_3_ nanorod bundles, achieving an energy density of 27.3 Wh kg^−1^ and capacitance retention of 82.6% after 5000 cycles [16]. Similarly, Mineo et al. reported a specific capacitance of 632 F/g and an impressive energy density of 90 Wh/kg using WO_3_ nanorods paired with graphene paper [17]. Further studies by Shinde et al. and Yao et al. confirmed the electrochemical viability of hydrothermally synthesized WO_3_ nanorods, reporting specific capacitances of 538 F/g and 319.26 F/g, respectively, alongside promising cycling stabilities [18,19]. These investigations highlight the substantial potential of WO_3_ as an electrode material. However, persistent issues related to its electronic conductivity and mechanical integrity during repetitive charge–discharge cycling underscore the need for further improvements [20]. One effective strategy to overcome these drawbacks involves the hybridization of WO_3_ with other electrochemically active materials, particularly cobalt oxide (Co_3_O_4_). As a spinel-structured TMO, Co_3_O_4_ exhibits high electrical conductivity, mixed valence states (Co^2+^/Co^3+^) that promote rich surface redox reactions, and excellent mechanical robustness. These characteristics make Co_3_O_4_ an ideal complement to WO_3_, providing an opportunity to synergistically enhance both ion transport and charge storage efficiency [6,21]. Several studies have demonstrated the outstanding pseudocapacitive performance of Co_3_O_4_-based electrodes. For example, Rabani et al. achieved 214 F/g with 94% retention over 5000 cycles using a Co_3_O_4_@CNF hybrid structure [22]. Similarly, Meher et al. demonstrated the excellent rate capability and long-term stability of ultralayered porous Co_3_O_4_, which delivered 548 F/g at 8 A/g [2]. Recently, J. Chen et al. reported a WO_3_@Co_3_O_4_ nanoarray heterostructure exhibiting specific capacitance of 1054 F/g at 0.5 A/g current, with cycling stability of 89% after 15,000 cycles [23]. These reports underscore the capacity of Co_3_O_4_ to contribute significantly to the development of high-performance SCs [24].

The rational design of the WO_3_@Co_3_O_4_ layered structure presents a compelling approach to address the individual limitations of each component. In such architectures, the faradaic reactions of WO_3_ are coupled with the surface-dominated redox activity of Co_3_O_4_, enabling the realization of electrodes that offer enhanced specific capacitance, rate capability, and cycling stability. The close interfacial contact between the two phases facilitates efficient electron transport while simultaneously providing improved structural integrity, which is critical for withstanding the mechanical stresses induced during repetitive cycling. In addition to compositional optimization, recent advances have highlighted the importance of morphology control in further boosting electrochemical performance. Tailoring the micro- and nanostructure of electrode materials can maximize the exposure of active sites, enhance electrolyte penetration, and minimize charge transport resistance. For example, Guan et al. reported a WO_3_ nanorod/rGO composite, synthesized through a one-pot hydrothermal process, that delivered 343 F/g at 0.2 A/g owing to improved electrical conductivity [25]. Similarly, Cai et al. demonstrated the role of strong interfacial interactions between WO_3_ and graphene nanosheets (GNS) in enhancing the capacitance of GNS–WO_3_ composites to 143.6 F/g at 0.1 A/g [7]. Zou et al. employed an electrodeposition strategy to fabricate WO_3_/PANI composites, where the morphology transitioned from nanoparticles to nanofibers as a function of aniline concentration, ultimately achieving 201 F/g at 1.28 mA/cm^2^ [26]. Parallel to these efforts, hybrid composites based on Co_3_O_4_ have also shown remarkable electrochemical behavior. Indumathi et al. developed CS-rGO-Co_3_O_4_ nanocomposites that exhibited a coulombic efficiency of 99% over 10,000 cycles and delivered an energy density of 23.6 Wh/kg at a power density of 734.1 W/kg, further reinforcing the potential of Co_3_O_4_ in high-performance energy storage systems [27]. Despite these promising developments, the practical performance of WO_3_@Co_3_O_4_ hybrid electrodes is often limited by non-ideal morphology, particle agglomeration, and poor electrolyte accessibility, which collectively restrict the utilization of active sites and hinder efficient charge transport. Addressing these issues requires precise control over the material’s morphology during synthesis, which can be effectively achieved through the use of directing agents. Despite significant progress in WO_3_-based and WO_3_/Co_3_O_4_ composite electrodes, the rational design of their nanostructure through controlled synthesis strategies remains a critical challenge. In particular, the role of surfactants in tailoring the morphology, particle size, and interfacial properties of such layered heterostructures has not been systematically explored. Moreover, establishing a clear correlation between surfactant-assisted structural modulation and electrochemical performance is still lacking. In this context, the present work focuses on the surfactant-assisted electrodeposition of WO_3_@Co_3_O_4_ layered electrodes using different structure-directing agents. By tuning the growth environment, a hierarchical porous architecture with reduced particle size is achieved, leading to enhanced ion transport and charge storage behavior. This study provides new insights into the design of high-performance layered oxide electrodes for supercapacitor applications. In this context, the strategic incorporation of surfactants and structure-directing molecules has emerged as a powerful approach to engineer electrode morphology. For instance, Morka et al. synthesized WO_3_/Ag_2_O nanocomposites via chemical bath deposition using various surfactants, where the WO_3_/Ag_2_O @Pluronic F-127 electrode exhibited an excellent specific capacitance of 960 F/g at 5 mV/s, outstanding cycling retention (135% after 2000 cycles), and a high energy density of 85 Wh/kg [28]. Similarly, Liu et al. employed sodium dodecyl sulfate (SDS) during the anodization of WO_3_, which not only guided the morphology but also induced a phase transformation, ultimately achieving a high volumetric capacitance of 1402.9 F/cm^3^ and exceptional cycling stability (106% retention after 10,000) [29]. Recent studies have highlighted that rational design of electrode materials, including heterostructure formation and nanostructure engineering, plays a crucial role in enhancing supercapacitor performance by improving ion diffusion, electrical conductivity, and electroactive surface area. Moreover, the integration of transition metal oxides and composite architectures has been widely explored to achieve superior capacitance, energy density, and long-term cycling stability. These advancements underline the importance of controlled synthesis strategies for next-generation energy storage materials [30,31,32,33].

In this work, we present a surfactant-assisted strategy for the fabrication of layered WO_3_@Co_3_O_4_ electrodes via sequential electrodeposition. The pristine sample prepared without surfactants is designated as W@Co, while the electrodes synthesized with hexamethylenetetramine (HMTA) and polyacrylic acid (PAA) as directing agents are denoted as H-W@Co and P-W@Co, respectively. The incorporation of these surfactants regulated nucleation and growth, yielding uniform and interconnected layered architectures with minimized agglomeration and improved electrolyte accessibility. Consequently, the surfactant-assisted electrodes (H-W@Co and P-W@Co) exhibited markedly enhanced electrochemical performance, including higher specific capacitance and excellent cycling stability, compared to the pristine W@Co electrode. Beyond performance improvements, this approach provides a simple and scalable pathway for engineering layered TMO electrodes on nickel foam, underscoring the pivotal role of morphology control in the advancement of next-generation energy storage systems.

## 2. Experimental Section

### 2.1. Materials

Sodium tungstate dihydrate (Na_2_WO_4_·2H_2_O, ≥99%), cobalt nitrate hexahydrate (Co(NO_3_)_2_·6H_2_O, ≥98%), sodium sulfate (Na_2_SO_4_, ≥99%), sodium hydroxide (NaOH, ≥98%), hydrogen peroxide (H_2_O_2_, 30%), nitric acid (HNO_3_, 65%), hexamethylenetetramine (HMTA, ≥99%), and polyacrylic acid (PAA, Mw ~250,000) were obtained from Sigma-Aldrich. Nickel foam (NF, thickness ~1.6 mm, porosity ~95%) was purchased from MTI Corp. (Richmond, CA, USA). All chemicals were used without further purification, and deionized (DI) water was employed in all solution preparations.

### 2.2. Fabrication of WO_3_@Co_3_O_4_ Layered Structures

NF was carefully pretreated prior to electrode fabrication to ensure a clean and oxide-free surface. The substrates were immersed in 2 M HCl and sonicated for 15 min to remove surface oxides, followed by sequential ultrasonication for 10 min in acetone, ethanol, and DI water. The cleaned foams were dried at 60 °C and immediately employed for deposition to avoid re-oxidation.

For the deposition of the WO_3_ base layer, a precursor solution was prepared by dissolving 25 mM Na_2_WO_4_·2H_2_O in 100 mL DI water. To this solution, 1 mL of 30% H_2_O_2_ was added, yielding a yellow peroxotungstate complex. A dilute HNO_3_ solution was then added dropwise at 45 °C until the pH stabilized at ~1.5, thereby ensuring precursor stability. After cooling to room temperature, the solution was used for electrodeposition. WO_3_ films were deposited on NF using a Biologic WBCS3000 electrochemical workstation (BioLogic Science Instruments, Seyssinet-Pariset, France) in a three-electrode system (NF as the working electrode (1 × 1 cm^2^), Pt wire as counter, and Ag/AgCl reference). Cyclic voltammetry (CV) was performed within a potential window of ±1.0 V at 50 mV/s for 25 cycles and optimized to yield uniform and adherent WO_3_ coatings. The resulting electrodes were rinsed with DI water and ethanol and dried at 60 °C.

**Figure 1:**
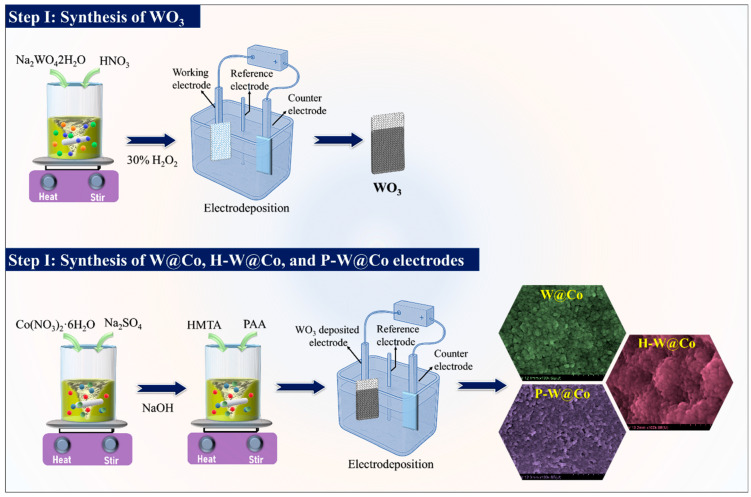
Schematic representation of the synthesis way for pristine and modified WO_3_@Co_3_O_4_ electrodes.

Following the WO_3_ deposition, a 4 overlayer was electrodeposited to form the WO_3_@Co_3_O_4_ layered structures. The electrolyte was prepared by dissolving 0.20 M Co(NO_3_)_2_·6H_2_O, 0.20 M Na_2_SO_4_, and 0.08 M NaOH in 100 mL DI water with continuous stirring. To investigate the role of structure-directing agents, HMTA or PAA were added at optimized concentrations. Electrodeposition was carried out in the same three-electrode setup within −1.2 V to +1.0 V vs. Ag/AgCl for 25 cycles, enabling controlled growth of Co_3_O_4_ nanostructures on the WO_3_ underlayer. After deposition, the WO_3_@Co_3_O_4_ NF were rinsed, dried at 80 °C overnight, and annealed in air at 400 °C for 2 h. For clarity, the synthesized samples are abbreviated as W@Co (WO_3_@Co_3_O_4_ prepared without surfactant), H-W@Co (WO_3_@Co_3_O_4_ prepared using HMTA as the surfactant), and P-W@Co (WO_3_@Co_3_O_4_ prepared using polyacrylic acid (PAA) as the surfactant). Figure 1 shows schematic of pristine and surfactant-assisted WO_3_@Co_3_O_4_ layered structures synthesis process.

### 2.3. Sample Characterization and Electrochemical Measurements

The phase composition and crystallinity of the pristine and surfactant-assisted WO_3_@Co_3_O_4_ electrodes were examined via XRD (PAN-analytical, Cu-Kα source), allowing clear identification of crystalline phases and structural integrity. Surface architecture and elemental distribution were studied using FE-SEM (S4800, HITACHI, Chiyoda, Tokyo, Japan) combined with EDS (energy-dispersive X-ray spectroscopy) analysis. Prior to imaging, a thin platinum layer was sputtered onto the samples to minimize charging effects. FE-SEM images revealed detailed surface textures, while EDS confirmed the elemental presence and dispersion. For insights into surface chemistry and oxidation states, X-ray photoelectron spectroscopy (XPS, K-Alpha, Thermo Scientific, Eastleigh, Dartford, UK) was employed, enabling evaluation of elemental valency. Electrochemical assessments were carried out using a Biologic WBCS3000 battery cycler (BioLogic Science Instruments, Seyssinet-Pariset, France) in a three-electrode setup, where the pristine and surfactant-assisted WO_3_@Co_3_O_4_ electrode acted as the working electrode, with platinum and Ag/AgCl as the counter and reference electrodes, respectively. A 2 M KOH solution served as the electrolyte to probe capacitance, charge–discharge characteristics, and cycling stability.

## 3. Results and Discussions

### 3.1. XRD Elucidation

**Figure 2:**
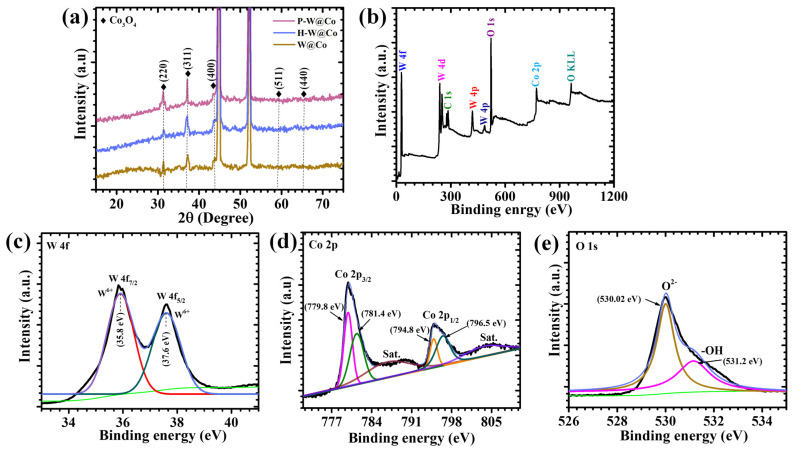
(**a**) XRD pattern of all electrodes; (**b**) XPS survey spectrum: High-resolution XPS spectrum of (**c**) W 4f (**d**) Co 2p, and (**e**) O 1s of P-W@Co electrode.

The XRD patterns for the three electrodes—W@Co, H-W@Co, and P-W@Co—are displayed in Figure 2a. All samples exhibit sharp and well-defined diffraction peaks that correspond exclusively to the cubic phase of Co_3_O_4_ (JCPDS No. 42-1467), indicative of its crystalline nature. The characteristic Co_3_O_4_ reflections at 2θ values near 31.41°, 37.02°, 44.2°, 59.4°, and 65.4° can be indexed to the (220), (311), (400), (511), and (440) planes, respectively. In contrast, the broad and featureless diffraction region associated with WO_3_ confirms its predominantly amorphous state within these samples, as no distinct crystalline peaks related to monoclinic WO_3_ were observed. The results indicate that the WO_3_ layer exhibits predominantly amorphous characteristics, which is commonly observed in electrodeposited WO_3_ films due to the rapid nucleation and low-temperature growth conditions during deposition. Compared with crystalline WO_3_, amorphous WO_3_ possesses a more disordered atomic arrangement with abundant unsaturated sites and open diffusion pathways, which can facilitate faster ion transport and improved electrolyte accessibility during electrochemical reactions. Such structural features are advantageous for pseudocapacitive charge storage, as they allow efficient redox reactions without significant structural strain. However, amorphous materials may exhibit relatively lower intrinsic conductivity than their crystalline counterparts. In the present WO_3_@Co_3_O_4_ layered system, the presence of the Co_3_O_4_ component helps enhance charge transfer and compensates for this limitation, resulting in improved electrochemical performance and stable cycling behavior. For the P-W@Co electrode, the Co_3_O_4_ peaks are notably sharper and exhibit increased intensity compared to the W@Co sample, reflecting enhanced crystallinity induced by the presence of PAA during synthesis. This surfactant promotes controlled nucleation and uniform grain development, which facilitates improved electron transport and structural integrity beneficial during electrochemical cycling. Conversely, the H-W@Co electrode shows discernible broadening of the Co_3_O_4_ diffraction peaks. Overall, the XRD analysis confirms the coexistence of crystalline Co_3_O_4_ and amorphous WO_3_ phases consistently across all electrode samples [15,19].

### 3.2. XPS Analysis

XPS is a powerful tool to probe the surface chemistry and oxidation states of electrode materials. To unravel the origin of its superior electrochemical behavior, XPS was carried out on the most optimized electrode, P-W@Co. The comprehensive XPS survey spectrum of the synthesized P-W@Co electrodes, as presented in Figure 2b, unequivocally verifies the successful incorporation of WO_3_ and Co_3_O_4_ phases. The elemental survey further confirms the presence of W, Co, and O within the fabricated electrode, signifying the effective synthesis of the desired material. Furthermore, the corresponding high-resolution spectra are presented in Figure 2c–e. The W 4f region (Figure 2c) exhibits two well-defined peaks at ~35.8 eV and ~37.6 eV, corresponding to the W 4f_7/2_ and W 4f_5/2_ states of W^6+^. The existence of W^6+^ reveals a rich and stable oxidation-valence state, which is particularly beneficial as it creates extra redox-active sites and allows smooth W^6+^/W^5+^ transitions during cycling. This feature is directly linked to the high charge-storage capability of the electrode [20]. The Co 2p spectrum (Figure 2d) shows well-defined peaks at 779.8 eV and 781.4 eV corresponding to Co 2p_3/_. Meanwhile, another character peaks at 794.8 eV and 796.5 eV attributed to Co 2p_1/2_, together with shake-up satellites, confirming the spinel nature of Co_3_O_4_. The balanced coexistence of Co^2+^ and Co^3+^ species reflects a favorable electronic configuration that supports fast and reversible redox processes [8]. The O 1s spectrum (Figure 2e) displays a strong lattice oxygen peak at 530.02 eV, along with a significant contribution at 531.2 eV arising from defect oxygen and surface-adsorbed oxygen species. The high density of defect oxygen demonstrates that P-W@Co possesses a defect-rich surface, which improves electrolyte penetration and accelerates reaction kinetics. Taken together, the XPS results reveal that P-W@Co uniquely combines stable W oxidation states, a balanced Co^2+^/Co^3+^ ratio, and abundant defect oxygen species, producing a highly active surface environment. These features provide a clear explanation for its superior electrochemical performance and firmly establish P-W@Co as the most effective electrode in this study [22].

### 3.3. Morphological and Elemental Composition

**Figure 3:**
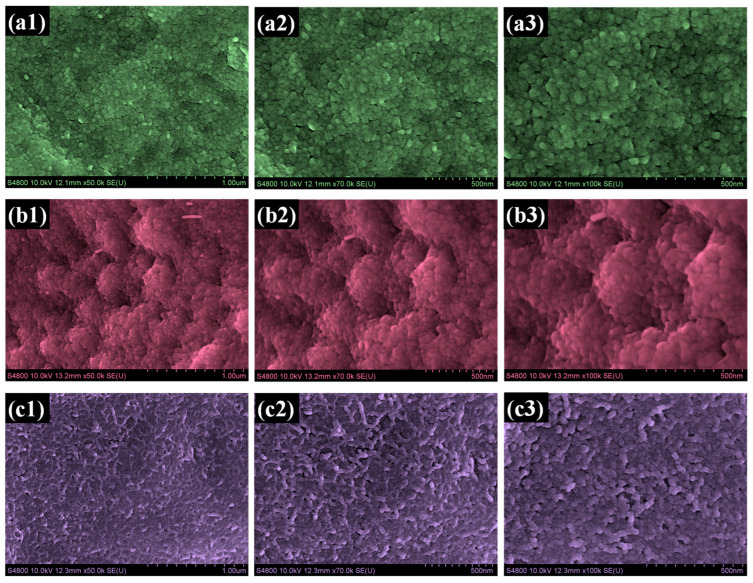
Top-view FE-SEM images of (**a1**–**a3**) W@Co, (**b1**–**b3**) H-W@Co, and (**c1**–**c3**) P-W@Co samples at different magnifications.

To elucidate the influence of surfactants on structural evolution, FESEM was employed to analyze the pristine and modified WO_3_@Co_3_O_4_ electrodes, and the resulting images are shown in Figure 3(a1–c3). The images demonstrate a clear modification in structural features depending on the presence and type of surfactant used during electrodeposition. The pristine W@Co electrode exhibits a compact granular morphology, as depicted in Figure 3(a1–a3). The particles are tightly packed and merge into a dense, uniform coating that completely blankets the nickel foam. Only a few shallow voids are visible, which indicates limited surface roughness and minimal open channels for electrolyte access. This morphology provides a smooth appearance but restricts the availability of ion-accessible pathways. In the H-W@Co electrode, prepared using HMTA, the surface evolves into cauliflower-like clusters, as illustrated in Figure 3(b1–b3). These clusters are composed of aggregated fine particles that fuse into irregular spherical features. The surface appears rougher compared to W@Co, with noticeable interparticle gaps and narrow pores distributed across the structure. However, some regions still show particle agglomeration, which partially reduces surface uniformity and connectivity. The P-W@Co electrode in Figure 3(c1–c3), fabricated with PAA, displays a markedly different architecture. The surface is built from uniformly dispersed interconnected nanogranules, which link together to form an open and continuous porous framework. The distribution of pores is more homogeneous, and the texture is finer and more evenly organized compared to both W@Co and H-W@Co. This structure minimizes aggregation and provides a highly interconnected network with abundant open channels across the surface [34,35]. When comparing the three samples, a clear morphological progression can be seen. The pristine W@Co forms a smooth and compact film, whereas the HMTA-assisted sample produces clustered and irregular features, and the PAA-assisted electrode develops into a porous framework of interconnected nanogranules. Among them, the morphology of P-W@Co stands out because of its uniformity and openness, offering extensive active surface exposure and efficient electrolyte accessibility. In addition to morphological observations, the average particle size of the synthesized electrodes was estimated from the FESEM images. The W@Co sample exhibits an average particle size of approximately 40.8 nm, which decreases to 35.21 nm for H-W@Co and further to 27.15 nm for P-W@Co. This progressive reduction in particle size can be attributed to the role of surfactants in controlling nucleation and growth during electrodeposition. The smaller particle size observed in the P-W@Co electrode leads to a higher surface-to-volume ratio and increased availability of electroactive sites, which facilitates enhanced ion diffusion and charge transfer kinetics. This structural advantage strongly correlates with the superior electrochemical performance observed for the P-W@Co electrode. These structural qualities directly support the superior electrochemical performance observed for P-W@Co.

**Figure 4:**
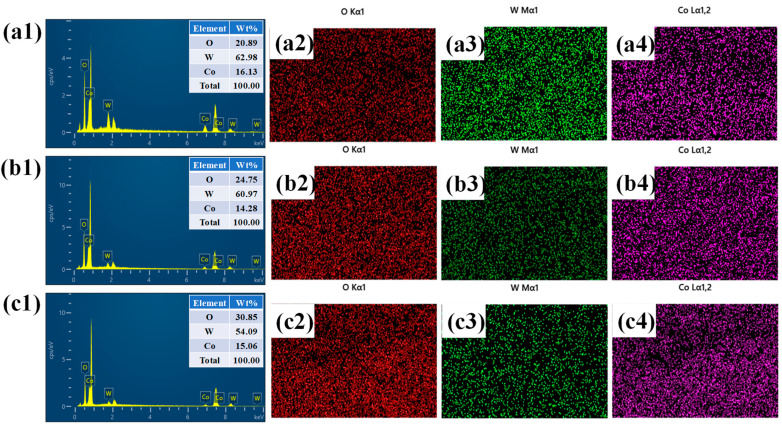
EDS and mapping analysis of (**a1**–**a4**) W@Co, (**b1**–**b4**) H-W@Co, and (**c1**–**c4**) P-W@Co samples.

Elemental composition analysis of the layered WO_3_@Co_3_O_4_ electrodes, including the pristine W@Co and the surfactant-assisted H-W@Co and P-W@Co samples, was performed using energy-dispersive EDS. The corresponding spectra shown in Figure 4(a1–c4) confirm the dominant presence of tungsten, cobalt, and oxygen elements, which verifies the successful sequential deposition of WO_3_ and Co_3_O_4_ onto the NF substrate. The insets in Figure 4(a1–c1) present the elemental weight percentages, further supporting the expected stoichiometric composition of the hybrid films. To gain deeper insight into spatial distribution, EDS elemental mapping was carried out for each electrode. The mapping images displayed in Figure 4(a2–c3) clearly reveal that tungsten, cobalt, and oxygen are uniformly dispersed throughout the electrode surface. No elemental deficiency was observed, indicating homogeneous nucleation and growth across the entire substrate. This uniform elemental spread confirms that both pristine and surfactant-assisted electrodes possess well-integrated and compositionally stable architectures. Such a consistent distribution of tungsten, cobalt, and oxygen is particularly significant, as it not only reflects the reliability of the electrodeposition strategy but also provides a strong structural foundation for the observed electrochemical performance of these electrodes.

### 3.4. Electrochemical Analysis

**Figure 5:**
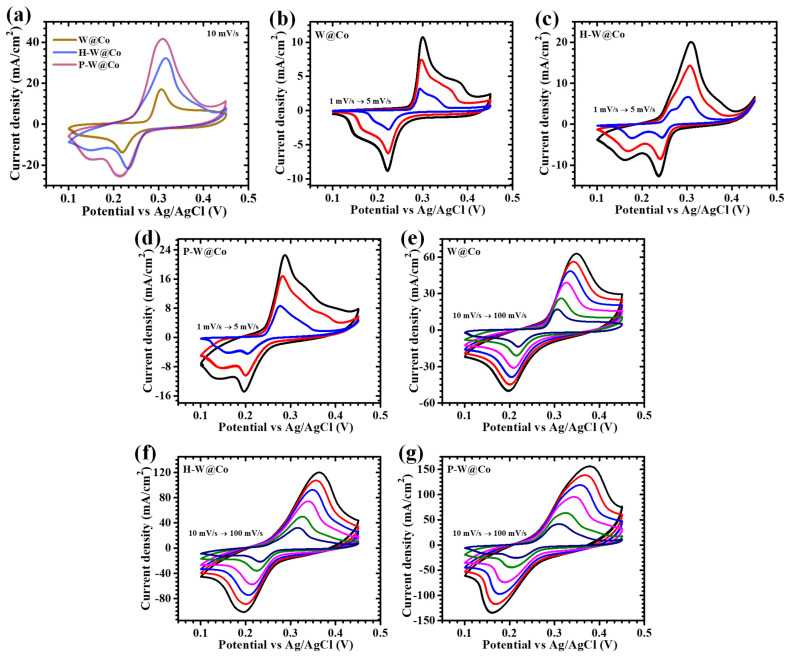
(**a**) Cyclic voltammetry of all electrodes at a scan rate of 10 mV/s: CV at a low scan rate from 1 to 5 mV/s for (**b**) W@Co, (**c**) H-W@Co, and (**d**) P-W@Co; CV at a high scan rate from 10 to 100 mV/s for (**e**) W@Co, (**f**) H-W@Co, and (**g**) P-W@Co electrodes.

The electrochemical performance of the W@Co, H-W@Co, and P-W@Co electrodes was precisely examined through analytical techniques including cyclic voltammetry (CV), galvanostatic charge–discharge (GCD), and electrochemical impedance spectroscopy (EIS), all within a three-electrode system employing 2 M KOH as the electrolyte. Figure 5a illustrates the CV profiles recorded at a constant scan rate of 10 mV/s over a potential window of 0.1 to 0.45 V for all three samples. This comprehensive investigation into the effects of HMTA and the PAA unveils how these additives distinctly influence the electrodeposition process, thereby modulating the electrochemical behavior and functional attributes of the resulting electrodes findings which are critical for high-performance energy storage applications. To thoroughly compare and characterize their electrochemical behavior, CV measurements were also conducted over a broad range of scan rates, from slow scans of 1 to 5 mV/s (Figure 5b–d) to more rapid scans of 10 to 100 mV/s (Figure 5e–g). The observed CV curves demonstrate evident redox peaks accompanied by a distinct deviation from ideal rectangular shapes, indicative of pseudocapacitive behavior governed primarily by reversible faradaic reactions. Specifically, the oxidation peaks centered around ~0.3 V and the corresponding reduction peaks near ~0.2 V underscore the robust and reversible electrochemical activity intrinsic to the WO_3_@Co_3_O_4_ layered architecture. These peaks corroborate the simultaneous reversible redox reactions occurring at the electrode interface, revealing exceptional electrochemical kinetics. Within the alkaline environment of the KOH electrolyte, the WO_3_@Co_3_O_4_ layered electrodes benefit from two interrelated faradaic processes: redox transitions in the cobalt oxide layer and the tungsten oxide layer. This synergistic mechanism substantially elevates the overall pseudocapacitive performance. The redox reactions can be briefly expressed as in Equations (1) and (2) [36,37]:

(1) $${WO}_{3}+xK^{+}+{xe}^{-}↔K_{x}{WO}_{3}$$

(2) $${Co}_{3}O_{4}+{OH}^{-}+H_{2}O+e^{-}↔3CoOOH↔{CoO}_{2}+H_{2}O+e^{-}$$

The CV responses captured at various scanning rates (Figure 5e–g) maintain well-defined shapes with minimal peak potential shifts, reflecting rapid charge transfer and low electrode polarization. The augmentation of the CV enclosed area with increasing scan rate signals enhanced electrochemical activity, which can be attributed to the thinning of the diffusion layer at the electrode surface, thereby enabling a greater faradaic reaction and resulting in higher current densities. Among the studied samples, P-W@Co demonstrated a notably larger redox peak current and significantly expanded CV area compared to W@Co and H-W@Co, indicative of its superior electrochemical charge storage capacity. This enhanced performance is attributed to the unique interconnected granular nanostructure adopted by PAA during electrodeposition. The surfactant effect of PAA effectively mitigates nanoparticle agglomeration, promoting uniform dispersion and the formation of tubular nanoparticle assemblies. This well-ordered structure not only offers abundant electroactive sites but also ensures efficient electron and ion transport pathways, thereby substantially elevating electrode kinetics and overall performance [38].

**Figure 6:**
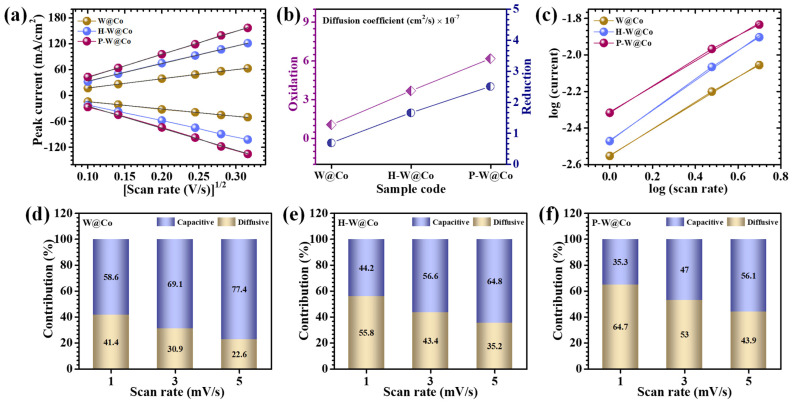
(**a**) Plot of peak current vs. (scan rate)^1/2^; (**b**) graphical presentation of calculated diffusion coefficient values at 10 mV/s scan rate; (**c**) plot of *log*(*i*) against the *log*(*ϑ*) for b value; capacitive and diffusion-controlled processes at different scan rates for (**d**) W@Co, (**e**) H-W@Co, and (**f**) P-W@Co electrodes.

Subsequently, CV responses acquired at multiple scan rates were subjected to rigorous analysis to elucidate the intricate redox dynamics supporting the electrode processes. Figure 6a defines the linear dependency of anodic and cathodic peak currents (*i_p_*) on the square root of the scan rate (*v*^1/2^), affirming diffusion-controlled electrochemical kinetics across all electrode constructs. The progressive augmentation of peak currents related to elevated scan rates corroborates the efficient charge transfer mechanisms operative within the system. To quantitatively probe the diffusional transport phenomena and electron transfer kinetics characterizing pristine and surfactant-modulated WO_3_@Co_3_O_4_ electrodes, the diffusion coefficients were methodically derived utilizing the Randles–Sevcik formalism (3) [39]:

(3) $$D^{1/2}=\frac{i_{p}}{2.69\times 10^{5}\times n^{3/2}\times A\times C\times v^{1/2}}$$

wherein *n* signifies the electron stoichiometry per redox reaction, *A* represents the electroactive surface area, *C* denotes the bulk concentration of the redox species, *D* is the diffusion coefficient, and *v* corresponds to the scan rate. The diffusion coefficients determined at a scan rate of 10 mV/s are compiled in Table 1 and graphically represented in Figure 6b. Significantly, the P-W@Co electrode demonstrated superior diffusivity for both oxidative and reductive processes compared to its counterparts. This enhancement is principally ascribed to the structural and physicochemical modifications imparted by the polymeric surfactant during synthetic elaboration. Variations in surfactant chemistry cause distinct morphological architectures, modulate the electrode/electrolyte interfacial energetics, and facilitate superior ionic mobility [40]. The elevated diffusion coefficient obtained for the P-W@Co system characterizes the optimized ion transport kinetics conducive to heightened electrochemical responsiveness. Conversely, pristine W@Co and H-W@Co displayed comparatively diminished diffusion coefficients, potentially owing to their denser morphologies and partial nanoparticle aggregation that obstruct active redox sites and impose diffusion barriers. Although H-W@Co evidenced moderate kinetic enhancement over the unmodified W@Co, it fails to attain the exceptional diffusion and transport properties realized through PAA surfactant control. This disparity underscores the profound impact of surfactant-assisted morphology control on enhancing electrode performance metrics.

For the intrinsic charge storage mechanisms governing the pristine W@Co, H-W@Co, and P-W@Co systems, a comprehensive kinetic assessment was performed by analyzing the peak current (*i_p_*) dependence on scan rate (*v*) through a power-law equation. This approach constitutes a rigorous framework for discriminating between surface-controlled capacitive phenomena and diffusion-limited faradaic processes inherent to electrochemical interfaces. The empirical relationship can be expressed as in Equations (4) and (5) [41]:

(4) $$i=i_{cap}+i_{diff}={av}^{b}$$

(5) $$logi=\log\left(a\right)+b\;log(v)$$

where *a* and *b* are experiential constants, with the exponent b serving as an analytical parameter reflecting the dominant charge storage regime. A b-value near 0.5 characterizes diffusion-governed intercalation processes characteristic of battery-type electrodes, while a b-value close to 1.0 signifies a capacitive mechanism dominated by surface adsorption and electric double-layer effects. Intermediate b-values (0.5 < b < 1.0) are indicative of hybrid pseudocapacitance, commonly observed in supercapacitance and supercapattery materials [42]. As presented in Figure 6c and summarized in Table 1, the *log*(*i*) versus *log*(*v*) plot exhibits excellent linearity over the 1–5 mV/s interval, affirming the applicability of the power-law model. The b-values extracted for H-W@Co and P-W@Co fall within the hybrid range, suggesting a synergistic interplay between surface capacitive effects and diffusion-controlled bulk redox processes. Conversely, the pristine W@Co electrode displays a more capacitive dominant behavior with a b-value of approximately 0.81. Remarkably, the P-W@Co electrode demonstrates a b-value around 0.68, revealing an enhanced diffusion-dominated faradaic contribution. This finding is substantiated by Dunn’s method analysis, which deconstructs capacitive and diffusion-controlled current components across scan rates, corroborating the superior diffusion kinetics in the P-W@Co system. The dual charge storage mechanism, encompassing rapid surface redox transitions, enables the P-W@Co electrode to reconcile high power delivery with substantial energy density, a desirable characteristic for applications requiring rapid charge–discharge cycling without compromising overall capacity [43].

To dissect the individual roles of EDLC and diffusion-governed faradaic mechanisms in the comprehensive charge storage behavior, a rigorous kinetic study was undertaken leveraging CV data acquired at low scan rates. This analysis is grounded in the methodology initially developed by Dunn, which mathematically partitions the total current response (*i*(*V*)) at a given potential as a function of the scan rate (*v*) based on the relationship (6) [44,45]:

(6) $$i\;\left(V\right)=k_{1}v+k_{2}v^{1/2}$$

Within this equation, the linear component, *k*_1_*v*, corresponds to capacitive contributions arising predominantly from surface-confined processes such as electrostatic ion adsorption within the electric double layer at the electrode/electrolyte interface. Conversely, the term *k*_2_*v*^1/2^ characterizes the diffusion-limited faradaic processes, inherently linked to redox reactions occurring within the bulk active material. By plotting the experimentally measured current as a function of both *v* and *v*^1/2^, the capacitive (surface) and diffusion-controlled (bulk) charge storage components may be quantitatively resolved via linear regression analysis, wherein the intercept and slope of the fitted line correspond to *k*_1_ and *k*_2_, respectively. At a representative scan rate of 1 mV/s, the relative contributions of capacitive versus diffusion-controlled processes for pristine W@Co, H-W@Co, and P-W@Co were determined to be 58.6/41.4%, 44.2/55.8%, and 35.3/64.7%, respectively. These results compellingly demonstrate a progressive shift toward diffusion-dominated faradaic behavior with the incorporation of HMTA and subsequent polymeric surfactant PAA during the electrode fabrication procedure. The pristine electrode is characterized by dominant capacitive behavior, primarily attributable to its compact morphology and moderate electronic conductivity, which favor charge accumulation confined near the electrode surface rather than deep redox participation. In contrast, the P-W@Co electrode’s strategically engineered porous and hierarchical nanoarchitecture facilitates superior electrolyte permeation and bulk redox engagement, culminating in the highest diffusion-controlled fraction among the studied materials. The scan rate-dependent charge storage analysis, extending from 1 to 5 mV/s (Figure 6d–f for W@Co, H-W@Co, and P-W@Co), demonstrates a systematic increase in capacitive fraction associated with an appropriate decrease in diffusion-controlled storage. This phenomenon reflects inherent ion transport limitations: at lower scan rates, ample temporal allowance permits ions to access deeper electrode regions, promoting surface faradaic storage; contrarily, at elevated scan rates, sequential constraints inhibit ion penetration into the bulk, restricting charge storage primarily to surface or near-surface capacitive behavior [46].

**Figure 7:**
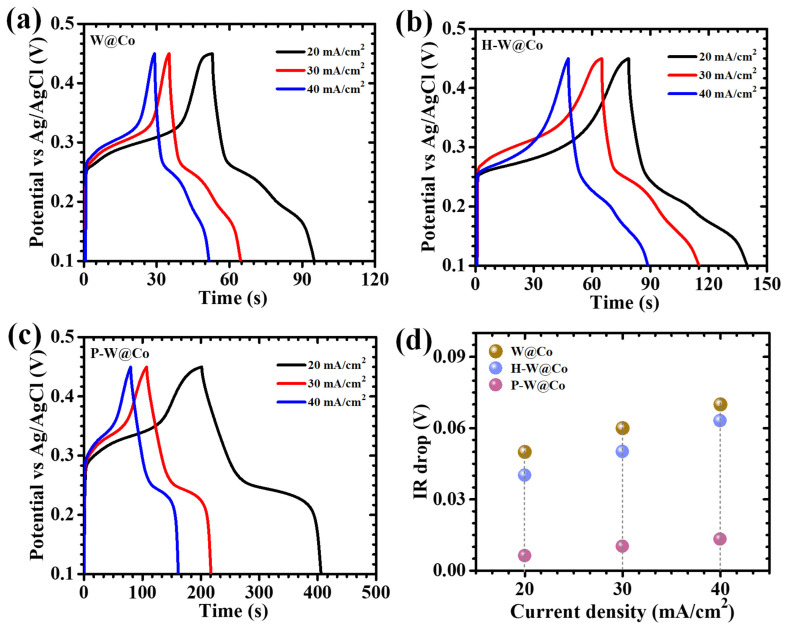
GCD curves of (**a**) W@Co, (**b**) H-W@Co, and (**c**) P-W@Co electrodes at different current densities (20–40 mA/cm^2^), and (**d**) plot of IR drop analysis.

In order to elucidate the effect of surfactant chemistry on the electrochemical properties of layered WO_3_@Co_3_O_4_ electrodes, a combination of galvanostatic charge–discharge (GCD) and electrochemical impedance spectroscopy (EIS) evaluations was undertaken. The GCD curves (Figure 7a–c), measured in the 0.1–0.45 V window at current densities between 20 and 40 mA/cm^2^, revealed typical nonlinear charge–discharge behavior with pronounced voltage plateaus across all electrode configurations, assertions of diffusion-influenced faradaic processes commonly found in battery-type storage electrodes [47]. Notably, the P-W@Co electrode exhibited the most marked nonlinearity, displaying a consistently smooth and extended discharge profile. This signals an enhanced pseudocapacitive response governed by the concerted action of surface-mediated redox reactions within the electrode nanostructure. Analysis of the GCD trends at varying current densities reinforced the conclusion that the P-W@Co sample, owing to its engineered morphology, is able to sustain prolonged discharge times and maximize charge storage relative to both pristine W@Co and H-W@Co. These electrodes maintained sign voltage features, correlated with redox transitions seen in CV curves across the entire current range, confirming the persistent dominance of pseudocapacitive charge storage. All electrodes showed nearly mirrored charge and discharge regions, emphasizing their high coulombic efficiency, suppressed polarization, and robust ion mobility. P-W@Co further distinguished itself by demonstrating minimal IR drop at discharge initiation as well as highly symmetric behavior regardless of current density. This reflects superior electronic conductivity, rapid ion transport, and the facilitation of highly reversible redox activity facilitated by the polymer surfactant-crafted framework. Charge transport resistance and kinetics were further interrogated by systematic IR drop analysis (Figure 7d), which revealed that all electrodes experience declining IR drop with reduced current density, indicative of diminished ohmic losses as diffusion dominates over resistive effects. Across all tested rates, the P-W@Co electrode consistently delivered the lowest IR drop values, providing direct evidence of its minimal internal resistance and optimized electrode–electrolyte interfacial contact. To rigorously assess electrochemical performance, the metrics of areal capacitance (C_A_), energy density (ED), and power density (PD) were calculated using equations specifically designed to handle the nonlinearities present in the GCD output for pseudocapacitive materials ((7)–(9)) [48,49]:

(7) $$C_{A}=\frac{I\times 2\times \int V\left(t\right)dt}{A\times {\left(∆V\right)}^{2}}$$

(8) $$ED=\frac{1}{2\times 3600}\;C_{A}\times {dV}^{2}$$

(9) $$PD=\frac{ED\times 3600}{T_{d}}$$

**Figure 8:**
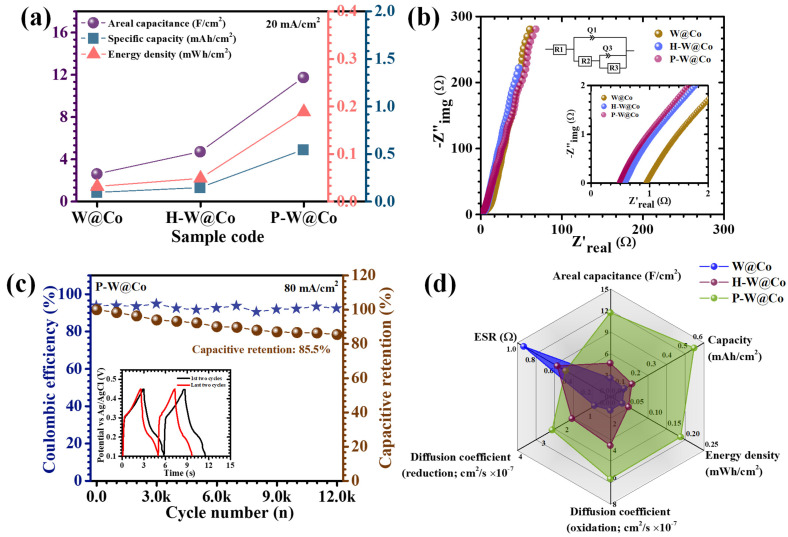
(**a**) Comparative assessment of areal capacitance, specific capacity, energy density of all electrodes; (**b**) Nyquist plot; (**c**) long-term cyclic stability test over 12,000 GCD cycles of P-W@Co sample (inset: first and last two GCD cycles); (**d**) radar plot.

In this calculation, *I* is the total current, time-integrated voltage response is *∫V*(*t*)*dt*, *A* is the effective electrode area (1 × 1 cm^2^), Δ*V* is available potential window, and *T* is the duration of the discharge, which are integrated into a cohesive experimental model. At a current density of 20 mA/cm^2^, the C_A_ values for W@Co, H-W@Co, and P-W@Co were determined as 2.56, 4.66, and 11.70 F/cm^2^, respectively (Table 2, Figure 8a), with the PAA-enabled P-W@Co electrode demonstrating indisputably superior charge storage. The striking performance improvement for the P-W@Co system is rooted in a synergy of interconnected advantages: (i) the emergence of a highly porous nanostructure produced by surfactant mediation, yielding dense arrays of accessible redox sites; (ii) the attainment of enhanced electronic transfer rates through delocalized electronic states and stabilized mixed W/Co valence; and (iii) short, unhindered pathways for ion mobility fostered by open, interlinked nanoparticle channels [50,51]. Capacitance and energy density profiles as a function of current density confirmed the typical decrease at higher rates, caused by incomplete OH^−^ diffusion into deeper electrode regions, which restricts redox participation to surface-proximal active sites while diminishing contributions from the electrode interior [52]. Remarkably, the P-W@Co electrode maintained 80% of its initial capacitance even under 40 mA/cm^2^ discharge, attesting to its high rate capability and the structural resilience engineered through polymeric surfactant design. Altogether, detailed GCD investigations underscore the profound advantages attributable to surfactant-directed nanoengineering in WO_3_@Co_3_O_4_ electrodes. The P-W@Co system demonstrates minimized resistive losses, maximized electroactive surface exposure, and unparalleled rapidity in charge transfer and ion transport.

EIS serves as an essential technique to dissect the intricate charge transport phenomena within electrode materials. The Nyquist plot, depicted in Figure 8b, displays a characteristic semicircle at high frequencies transitioning to an inclined line at lower frequencies, indicative of multiscale electrochemical processes. The intercept of the semicircle with the real axis (*X*-axis) represents the equivalent series resistance (R1), a composite metric embodying the electrolyte resistance, intrinsic resistance of the active material, and the resistance at the electrode–electrolyte interface. The diameter of the semicircle allows extraction of the charge transfer resistance, reflecting the kinetics of electrochemical reactions at the interface [53]. To gain deeper insight into the electrochemical kinetics, the EIS data were fitted using an equivalent circuit model consisting of R1 + Q1/(R2 + Q3/R3). As compiled in Table 1, the P-W@Co electrode exhibits the minimal ESR value of 0.48 Ω, substantially lower than the values recorded for the pristine and HMTA-modified H-W@Co electrodes. The fitted parameters for different electrodes reveal a significant variation in charge transport behavior. The P-W@Co electrode exhibits the lowest charge transfer resistance (R3 = 3.772 Ω), compared to H-W@Co (13.82 Ω) and W@Co (23.89 Ω), indicating more efficient electron transfer at the electrode–electrolyte interface. Furthermore, the Q1 value, representing the interfacial capacitive behavior, increases from W@Co (0.003835 F.s^n^) to H-W@Co (0.01416 F.s^n^) and reaches the highest value for P-W@Co (0.03424 F.s^n^), suggesting enhanced electroactive surface area and improved electrode–electrolyte interaction. The relatively lower resistance and higher capacitive response of the P-W@Co electrode can be attributed to its reduced particle size and hierarchical porous structure, which facilitate faster ion diffusion and charge transfer processes. These results are consistent with the superior electrochemical performance observed for the P-W@Co electrode.

The long-term cycling stability of supercapacitor electrodes at elevated current densities is a critical parameter for evaluating their practical applicability. To examine this, the durability of the optimized P-W@Co electrode was systematically evaluated by subjecting it to continuous GCD cycling at 80 mA/cm^2^ for an extended sequence of 12,000 cycles. Figure 8c depicts the capacitance retention and coulombic efficiency trends as a function of cycling number. In the initial phase, a gradual increase in capacitance was observed, likely due to electrode activation and progressive unblocking of internal pores that enhanced electrolyte infiltration. Upon reaching an activated state, the electrode demonstrated remarkably stable behavior with only negligible capacitance decay over prolonged cycling. After completing 12,000 cycles, the P-W@Co electrode retained approximately 85.5% of its initial capacitance, corresponding to a modest degradation of roughly 14%, thereby illustrating exemplary long-term electrochemical robustness. This retention underscores the structural integrity and chemical resilience of the architecture, which is capable of enduring repetitive redox processes cycles without significant mechanical deterioration. The surfactant-assisted framework effectively absorbs volumetric changes during cycling, mitigating pulverization and preserving the integrity of redox-active sites. Complementing this, the coulombic efficiency remained consistently high throughout the entire test duration, maintaining 92.3% even after 12,000 cycles. Such exceptional reversibility confirms minimal parasitic side reactions and highly efficient charge compensation during the faradaic processes. The combination of sustained capacitance retention and near-ideal coulombic performance validates the electrochemical durability of the P-W@Co electrode and affirms its capability to deliver rapid, reversible redox transformations under strenuous high-rate cycling conditions. Collectively, these findings establish the P-W@Co system as a robust and reliable candidate for next-generation supercapacitor applications, offering both longevity and performance consistency under operational stress.

The radar plot illustrated in Figure 8d offers a clear multidimensional comparison of the key electrochemical parameters of areal capacitance, specific capacity, energy density, ion diffusion coefficient, and ESR for the W@Co, H-W@Co, and P-W@Co electrodes. This graphical assessment highlights the outstanding and well-balanced performance of the optimized P-W@Co electrode. The broad and symmetrical distribution of its features demonstrates the effective integration of high charge storage ability, efficient ion transport, and low internal resistance within a single advanced electrode design. The PAA-directed electrodeposition strategy offers distinct advantages over conventional synthesis approaches reported for WO_3_@Co_3_O_4_ systems. The presence of PAA plays a crucial role in regulating the nucleation and growth kinetics, leading to the formation of a uniform, hierarchical porous structure with reduced particle size. This structural modulation not only increases the effective surface area and electroactive sites but also improves ion diffusion pathways and charge transfer efficiency. Furthermore, the enhanced interfacial interaction between amorphous WO_3_ and crystalline Co_3_O_4_ contributes to improved electrical conductivity and cycling stability. These combined effects distinguish the present approach from previously reported systems and account for the superior electrochemical performance observed.

A comparative analysis of previously reported WO_3-_based and Co_3_O_4_-based electrode materials is presented in Table 3 [54,55,56,57,58]. It can be observed that although various electrode systems exhibit promising electrochemical performance, their capacitance and cycling stability are often limited by factors such as insufficient active site utilization, poor electrical conductivity, or structural instability during prolonged cycling. In comparison, the P-W@Co electrode developed in this work demonstrates enhanced electrochemical performance, which can be attributed to its optimized nanostructure, reduced particle size, and improved electrode–electrolyte interactions induced by the PAA-assisted synthesis strategy. Furthermore, the stable cycling behavior indicates good structural integrity of the electrode during repeated charge–discharge processes. These results clearly highlight the advantage of the present approach over previously reported systems.

## 4. Electrochemical Performance of Asymmetric Supercapacitor Device

**Figure 9:**
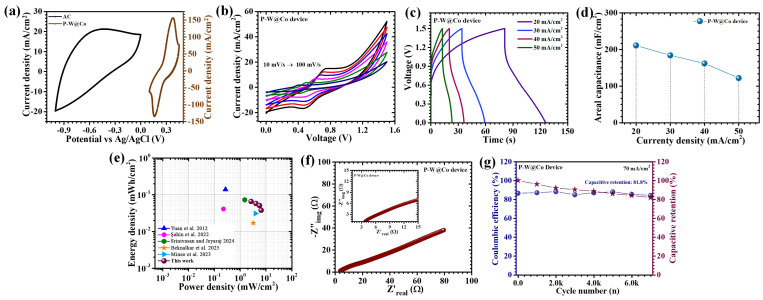
(**a**) CV responses of the AC and P-W@Co electrodes at 100 mV/s scan rate in three electrode system, (**b**) CV at variable scan rates (10–100 mV/s), (**c**) GCD curves at different current densities (20–50 mA/cm^2^), (**d**) rate capability, (**e**) Ragone plot [8,9,10,14,15], (**f**) Nyquist plot, and (**g**) stability test of P-W@Co asymmetric supercapacitor device.

In order to validate the practical potential of WO_3_@Co_3_O_4_ electrodes beyond the conventional three-electrode setup, an asymmetric supercapacitor device was fabricated and systematically investigated through comprehensive electrochemical analyses. The device architecture comprised the polymer-assisted P-W@Co electrode as the positive electrode, paired with activated carbon (AC) as the negative electrode, the latter being a standard material distinguished by its high specific surface area and superior double-layer capacitance characteristics. Both electrodes were directly deposited onto nickel foam current collectors, providing robust mechanical support and excellent electrical connectivity. A porous filter paper saturated with 2 M KOH electrolyte served as the separator, and the assembly was encapsulated within a sealed pouch cell to eliminate exposure to environmental contaminants and maintain operational stability over time. The electrochemical performance was rigorously evaluated through CV, GCD, and EIS techniques. To appropriately determine the operating voltage window of the assembled asymmetric supercapacitor device, CV measurements were first conducted for the individual electrodes in a three-electrode configuration. The P-W@Co electrode exhibited a distinct potential window ranging from approximately +0.1 to +0.45 V (vs. Ag/AgCl), reflecting its faradaic pseudocapacitive behavior. In contrast, the AC electrode displayed typical electric double-layer capacitive characteristics with stable operation in the potential range of −1.0 to 0.0 V, indicating its suitability as a negative electrode (Figure 9a). Further, CV profiles of the device (Figure 9b) recorded at various scan rates (10 to 100 mV/s) affirmed a stable operating potential window extending up to 1.5 V, which decisively surpasses the voltage limits of typical aqueous supercapacitor systems. This widened operational window is instrumental in enhancing the achievable energy density of the device. The CV curves preserved their distinct shapes across the scan rate range without notable distortion, and the current response scaled linearly with the increasing scan rate. Such electrochemical characteristics are indicative of highly reversible redox processes, efficient charge transport throughout the device, and strong compatibility at the electrode/electrolyte interface. The observed performance originates from the synergistic combination of the P-W@Co positive electrode, which primarily engages in pseudocapacitive faradaic reactions, and the AC negative electrode, which contributes rapid double-layer charge storage, thereby ensuring effective energy and power delivery. Further corroboration was provided by GCD measurements conducted over a range of current densities, which exhibited discharge curves with pronounced nonlinearity and well-defined pseudocapacitive features, characteristic of dominant faradaic charge storage processes (Figure 9c). At a current density of 20 mA/cm^2^, the device exhibited an areal capacitance of 211 mF/cm^2^, alongside an energy density of 0.066 mWh/cm^2^ and power density of 2.63 mW/cm^2^, attesting to the device’s capacity to balance high energy density with swift charge–discharge capability (Table 4). The nonlinear nature of these curves underscores the substantial contribution of the P-W@Co electrode’s pseudocapacitance, effectively complemented by the AC electrode’s fast double-layer capacitance. Even when operated at an elevated current density of 50 mA/cm^2^, the device maintained around 58% of its original capacitance, confirming its excellent rate performance (Figure 9d). The Ragone plot (Figure 9e) further illustrates the superior energy–power balance, revealing that the P-W@Co//AC device delivers a high areal energy density of 0.066 mWh/cm^2^ at a power density of 2.63 mW/cm^2^, which is better than or comparable to many previously reported asymmetric supercapacitors.

EIS measurements further illuminated the device’s charge transport dynamics. The Nyquist plots revealed a diminutive semicircle at high frequencies, corresponding to a very low equivalent series resistance (ESR) of 3.7 Ω (Figure 9f). This remarkably low ESR highlights the minimal intrinsic resistance afforded by the electrode materials, the facile ion transport across the separator, and the exceptional electrical conductivity within the electrode/electrolyte interface. Such electrochemical attributes ensure reduced losses and enhanced device efficiency. The long-term electrochemical stability, a critical factor for practical applications, was substantiated through prolonged GCD cycling at 70 mA/cm^2^ for 7000 cycles (Figure 9g). Impressively, the device retained 81.8% of its initial capacitance, exhibiting only marginal degradation under rigorous cycling conditions. Simultaneously, the coulombic efficiency consistently remained above 84%, underscoring the high reversibility of the faradaic reactions and the suppression of parasitic side reactions. The sustained performance can be directly attributed to the structural and chemical robustness of the P-W@Co electrode, whose evenly distributed, interconnected nanoparticle morphology maximizes electrolyte accessibility, inhibits agglomeration, and prevents surface area loss due to restacking. The slight capacitance decay observed during extended cycling can be attributed to the gradual loss of accessible electroactive sites, minor structural relaxation of the active material during repeated redox reactions, and possible ion trapping within the porous electrode structure. Nevertheless, the relatively high capacitance retention indicates that the P-W@Co layered architecture effectively maintains structural integrity and facilitates stable ion transport during prolonged charge–discharge processes. The surfactant optimization in the synthesis process plays a vital role in stabilizing this architecture. Consequently, the P-W@Co electrode excels not only in initial electrochemical activity but also in maintaining prolonged operational stability, thereby positioning itself as a compelling candidate for the development of next-generation energy storage technologies with outstanding durability and performance.

## 5. Conclusions

The present investigation successfully demonstrates that surfactant-assisted electrodeposition can effectively engineer the nanoscale architecture of WO_3_@Co_3_O_4_ layered electrodes to optimize their electrochemical performance for supercapacitor applications. The deployment of polyacrylic acid surfactant during synthesis resulted in a hierarchical porous morphology of the P-W@Co electrode that simultaneously maximizes accessible redox-active sites and electronic pathways while maintaining structural integrity during cycling. The amorphous WO_3_ matrix coupled with crystalline spinel Co_3_O_4_ synergistically facilitates robust pseudocapacitive charge storage dominated by diffusion-controlled faradaic processes and surface redox reactions. Extensive electrochemical evaluation, including rigorous three-electrode and practical asymmetric device testing, affirms the superiority of the P-W@Co electrode in terms of capacitance (11.70 F/cm^2^ at 20 mA/cm^2^), rate capability, and electrochemical stability. The asymmetric device architecture incorporating activated carbon negative electrodes expands the operational voltage window and balances fast double-layer charge storage with high-capacity faradaic reactions. Crucially, the device exhibits outstanding long-term cycling durability and high coulombic efficiency, underscoring the chemical and mechanical robustness imparted by the surfactant-engineered nanoarchitecture. This work highlights the critical role of surfactant-mediated morphology modulation in the development of next-generation layered transition metal oxide electrodes, advancing supercapacitor technology toward scalable, high-energy, high-power, and durable energy storage solutions.

## Figures and Tables

**Table 1:** Assessed diffusion coefficients, b-values, and ESR of all electrodes.

Sample Code	Diffusion Coefficient (cm^2^/s) × 10^−7^		b-Value	R1 (Ω)
	Oxidation	Reduction		
**W@Co**	1.03	0.68	0.8	0.93
**H-W@Co**	3.66	1.65	0.71	0.57
**P-W@Co**	6.15	2.5	0.68	0.48

**Table 2:** Calculation of energy storage parameters including areal capacitance, specific capacity, energy density, and power density values of W@Co, H-W@Co, and P-W@Co electrodes.

Sample Code	I (mA/cm^2^)	Areal Capacitance C_A_ (F/cm^2^)	Capacity (mAh/cm^2^)	Energy Density ED (mWh/cm^2^)	Power Density PD (mW/cm^2^)
**W@Co**	20	2.56	0.09	0.032	2.52
	30	2.52	0.089	0.031	3.88
	40	2.40	0.084	0.029	5.00
**H-W@Co**	20	4.66	0.140	0.049	2.20
	30	4.31	0.136	0.048	3.40
	40	4.29	0.123	0.043	4.31
**P-W@Co**	20	11.70	0.540	0.189	3.32
	30	9.44	0.438	0.153	5.01
	40	9.36	0.438	0.153	6.74

**Table 3:** Comparative summary of electrochemical performance of reported WO_3_-based and Co_3_O_4_-based electrode materials.

Sr. No.	Material	Electrolyte	Specific Capacitance	Current Density	Cycle Stability	Reference
**1.**	Co_3_O_4_	1M KOH	1.35 F/cm^2^	10 mA/cm^2^	90% (5000 cycles)	[54]
**2.**	Co_3_O_4_/NiCo_2_O_4_	6M KOH	3.53 F/cm^2^	1 mA/cm^2^	93.8% (10,000 cycles)	[55]
**3.**	WO_3_@NiWO_4_	3 M KOH	3.80 F/cm^2^	2 mA/cm^2^	96.63% (5000 cycles)	[56]
**4.**	WO_3_	1 M KOH	1.20 F/cm^2^	10 mA/cm^2^	91% (5000 cycles)	[57]
**5.**	MoO_3_/WO_3_/rGO	2 M KOH	1.17 F/cm^2^	10 mA/cm^2^	92.6% (2000 cycles)	[58]
**6.**	**PAA-WO_3_/Co_3_O_4_**	**2M KOH**	**11.7 F/cm^2^**	**20 mA/cm^2^**	**85.5%** **(12,000 cycles)**	**This Work**

**Table 4:** Energy storage performance of P-W@Co//AC asymmetric supercapacitor device.

Sample Code	I (mA)	CA (mF/cm^2^)	C (mAh/cm^2^)	ED (mWh/cm^2^)	PD (mW/cm^2^)
**P-W@Co device**	20	211	0.044	0.066	2.63
	30	184	0.038	0.058	4.07
	40	162	0.034	0.051	5.61
	50	122	0.025	0.038	6.49

## Data Availability

The data presented in this study are available on request from the corresponding author due to privacy reasons.
